# Highlights from the 6th International Society for Computational Biology Student Council Symposium at the 18th Annual International Conference on Intelligent Systems for Molecular Biology

**DOI:** 10.1186/1471-2105-11-S10-I1

**Published:** 2010-12-07

**Authors:** Christiaan Klijn, Magali Michaut, Thomas Abeel

**Affiliations:** 1 The Netherlands Cancer Institute, division of Molecular Biology, Amsterdam, The Netherlands; 2 The Bioinformatics Lab, Faculty of Electrical Engineering, Delft University of Technology, Delft, The Netherlands; 3 Donnelly Centre for Cellular and Biomolecular Research, University of Toronto, Ontario, Canada; 4 Banting and Best Department of Medical Research, University of Toronto, Ontario, Canada; 5 Department of Plant Systems Biology, VIB, Ghent University, Gent, Belgium; 6 Broad Institute of MIT and Harvard, Cambridge, MA, USA

## Abstract

This meeting report gives an overview of the keynote lectures and a selection of the student oral and poster presentations at the 6th International Society for Computational Biology Student Council Symposium that was held as a precursor event to the annual international conference on Intelligent Systems for Molecular Biology (ISMB). The symposium was held in Boston, MA, USA on July 9th, 2010.

## Introduction

The Student Council of the International Society for Computational Biology (ISCB) is a world-wide organization for students in computational biology and bioinformatics. The major aims of the Student Council are to organize events and facilitate networking opportunities for students. The main contribution of the Student Council is to nurture soft skills, such as working in a team, organizational and networking skills, to complement the normal academic program. Since its inception, the Student Council has organized an annual student symposium for the benefit of the student community. This year, the sixth ISCB Student Council Symposium was held in conjunction with the ISMB conference on July 9th. Over 110 delegates took part in this edition of the ISCB Student Council Symposium. The symposium opened with a scientific speed dating session to allow the delegates to get to know one another before the symposium kicked off. The program featured three keynote lectures, two partner presentations, twelve contributed student presentations and an extensive poster session with over 50 posters.

We were honoured to have three highly esteemed scientists deliver the keynote presentations. Gary Bader (University of Toronto, Canada) opened the scientific program with his presentation titled: ‘Predicting protein-protein interaction networks from the genome.’ The afternoon session was initiated by Larry Hunter (University of Colorado, Denver, USA) with a presentation titled ‘Thinking Big: How Artificial Intelligence and Molecular Biology can transform each other’. The final keynote lecture of the day was conducted by David Altshuler (The Broad Institute of MIT and Harvard, Boston, USA) and was titled ‘Genomic Variation and the Inherited Basis of Common Disease’

This year we welcomed two talks by our industrial and research partners. Katrina Pavelin of the European Bioinformatics Institute (Cambridge, UK) gave an overview of training and career opportunities at EMBL-EBI. Joeri van der Velde of the University of Groningen (The Netherlands) introduced the Netherlands Bioinformatics Center (NBIC) and presented MOLGENIS, an environment for rapid generation of extensible software platforms for genotypes and phenotypes.

## Proceedings

This year we received almost one hundred submissions from students to present their work at the symposium. These submissions were peer-reviewed by 30 independent reviewers from who the program committee selected 12 for oral presentation. An additional 55 abstracts were accepted to be presented in a poster. The twelve oral presentations fell into three main fields of research: Proteins, (Epi)Genomics and Bioinformatics of Health and Disease. Each of these topics featured a block of four presentations. Here we briefly discuss the presentations that are featured in this special proceedings issue.

### Proteins

Bioinformatics has been used to study proteins and protein interactions for a long time. One of the main questions in protein bioinformatics has been how to spatially model interactions between different proteins. Vanhee et al. [1] presented a novel approach that does not use whole protein structures for predicting interactions, but instead focuses on the interaction modelling of fragments. This approach showed promise in being able to correctly predict many interactions, possibly simplifying the search for novel interactions. Li et al. [2] showed a different perspective on protein interactions. They modelled proteins in a crowed environment, which is the natural state in a cell, and were able to predict striking effects on protein-protein binding for both high and low affinity binding partners using their macromolecular crowding model.

The prediction of the three dimensional structure of a protein is another large field in protein bioinformatics. As the structure of a protein influences much of its function, an accurate estimation of its spatial conformation is essential. Shah et al. [3] presented a novel approach to find similarities between protein structures that are not necessarily evident from the amino acid sequences. In their feature-based search they found that the incorporation of secondary structures sped up the alignment of similar structure while remaining very accurate. Finding structurally similar proteins is an important endeavour. Finding the exact sites where the catalysed reactions take place is equally important. Xin et al. [4] presented a structure-based kernel approach to find novel catalytic residues in protein sequence. Their approach out-performed existing methods and could be used to associate mutations implicated in heritable disease to specific catalytic activities of the mutated proteins.

### (Epi)Genomics

The field of (Epi)Genomics has exploded in scope in the last 10 to 15 years. The invention of the DNA micro array technique enabled large-scale investigations into gene expression, DNA structural changes, single-nucleotide variations in the human population and many other genome-wide assays. With the advent of new high throughput sequencing methods, the amount of data associated with studies into genomics has risen exponentially. The field of genomics data analysis will have to face this flood of new data [5].

With the newly acquired possibility to identity genome-wide patterns of chromatin modifications, analysis of genome-wide profiles of different modifications in the same samples poses an interesting challenge. Larson et al. [6] opened the (Epi)Genomics session with a new Hidden Markov Model based algorithm to find multi-gene domains from integrated ChIP-seq experiments. By combining the epigenetic profiles obtained from ChIP-seq of five different histone modification marks they were able to identify large scale organization of gene clusters.

Single Nucleotide Polymorphisms (SNPs) have replaced old genomic markers in large studies to find genomic locations associated with disease. These Genome Wide Association Studies (GWAS) typically investigate a disease in a large case-control cohort. Surendran et al. [7] reported on a GWAS that compared 14,000 cases and 3,000 shared controls in which they found 53 new loci associated with seven common diseases. By using only the non-synonymous SNPs they were able to extract more associations than the original studies reported. Even if an informative SNP has been associated with a disease, finding the causative variation is still a difficult problem. Since many SNPs are co-inherited with neighbouring SNPs defining the actual nucleotide responsible for the disease is a difficult task. Macintyre et al. [8] presented a novel algorithm to predict causative SNPs by focusing on the SNPs that disrupt transcription factor binding sites.

With the influx of data as a result of the development of high throughput sequencing technologies comes the substantial task of making sense of it all. Using the data that is a result of RNA sequencing experiments Behr et al. [9] developed a novel gene finding algorithm, mGene.ngs. They showed that their algorithm is more accurate than previously developed approaches and has the added benefit of predicting alternative transcripts.

### Bioinformatics of Health and Disease

Computational biology and bioinformatics have many applications in the field of health and disease. In this session, examples of the high diversity of possible studies were given. The presentations covered various topics, namely: metabolic modelling in heart disease, chemo informatics, anti-gen prediction and vaccine design. Hettling et al. [10] presented on applying a ‘sloppy’ modelling approach to metabolic data of the Creatine Kinase (CK) system in heart muscle. Using their approach, it was confirmed that CK acts as an energy buffer in the energy transport system of the heart muscle. Magariños et al. [11] continued with a description of tools implemented in the Tropical Disease Targets Database (tdrtargets.org). Using various features of novel chemical compounds they were able to make predictions for over 435,000 compounds to act on more than 3,500 clinically relevant targets. Carmona et al. [12] continued on the subject of pathogens in tropical disease. In this case they use novel approaches to predict potentially useful peptides useful as low cross-reactive antigens in diagnosis and treatment. They were successful in prioritizing peptides that were specific to positive serum samples. Banton et al. [13] wrapped up the proceedings part of the conference with a presentation on a bioengineering approach for vaccine design for the Ebola virus. Using mathematical models for predicting immune response efficacy and structure based epitope identification they were able to specify a peptide to use in Ebola vaccination.

### Posters

Another 55 abstracts were presented in the form of a poster presentation. From these presentations a winner and a runner-up prize for best poster presentations was selected by collecting ballots from the attendees as well as judgment by an independent jury. Unfortunately, the winner declined to have the abstract published in these proceedings. The runner-up for best poster presentation was McDowall et al. [14] who reported on prediction of human protein-protein interactions.

## Conclusions

Given the large number of abstracts submitted to the Student Council Symposium we were able to provide three high-quality student presentation sessions as well as an active and highly interactive poster session.

## The future

The next Student Council Symposium is planned to be held together with ISMB/ECCB 2011 in Vienna. For information on the Student Council and other events we organize for students in computational biology and bioinformatics, please see our website: http://www.iscbsc.org.
